# Effects of morphine and NeuroAid on the expression levels of GluN2A and GluN3A in the hippocampus and striatum of rats

**DOI:** 10.22038/ijbms.2021.52004.11787

**Published:** 2021-04

**Authors:** Katayoun Heshmatzad, Mohammad Nasehi, Salar Vaseghi

**Affiliations:** 1 Cognitive and Neuroscience Research Center (CNRC), Amir-Almomenin Hospital, Tehran Medical Sciences, Islamic Azad University, Tehran, Iran.; 2 Department of Cognitive Neuroscience, Institute for Cognitive Science Studies (ICSS), Tehran, Iran

**Keywords:** Glutamate, Hippocampus, Morphine, NeuroAid, Striatum

## Abstract

**Objective(s):**

NMDA glutamatergic receptors are heteromeric receptors with various subunits. GluN2A and GluN3A subunits specify the functional heterogeneity of NMDA receptors. These subunits play a key role in the induction of LTP and synaptic plasticity. Note that, the function of NMDA subunits has interaction with the mechanism of morphine. On the other hand, NeuroAid is a Chinese traditional medicine with neuroprotective and anti-apoptotic effects. In this study, we aimed to investigate the effect of morphine and NeuroAid on expression levels of GluN2A and GluN3A in the hippocampus and striatum of rats.

**Materials and Methods:**

Morphine sulfate (increasing doses) and NeuroAid (2.5 mg/kg) were injected intraperitoneally. Real-time PCR was used to assess gene expression.

**Results:**

The results showed that morphine increased the expression of GluN2A in the hippocampus and striatum, while NeuroAid increased the expression of both genes in the hippocampus and decreased the expression of GluN3A in the striatum. NeuroAid increased the expression of GluN3A in the hippocampus and GluN2A in the striatum of morphine-addicted rats.

**Conclusion:**

NeuroAid may have interaction with the effect of morphine on glutamatergic neurotransmission; however, this study is innovative and novel, thus, further studies are needed to better understand the effect of NeuroAid and morphine on hippocampal and striatal glutamatergic neurotransmission.

## Introduction

NMDA (N-Methyl-D-aspartate) subtype of glutamate receptors (GluNs) is a heteromeric receptor with various subunits including GluN1, GluN2A-D, and GluN3A-B that specify excitatory postsynaptic currents (EPSC) amplitudes (1). GluN1 is the obligatory subunit, while GluN2 and GluN3 specify the functional heterogeneity of NMDA receptors (2). The normal function of the NMDA receptor is crucial for synaptic plasticity, learning, memory, and information storage; while NMDA receptor dysfunction leads to cognitive and psychiatric disorders such as epilepsy, mental retardation, and schizophrenia (3, 4). The GluN2A:2B ratio has a major role in the regulation of decay kinetics of GluNs EPSCs and affects synaptic plasticity via modulating LTP (long-term potentiation) and LTD (long-term depression) (5). GluN2 subunits are phosphorylated by activity-dependent protein kinases including PKA (Protein kinase A - serines in GluN1, GluN2A, and GluN2C), PKC (Protein kinase C - serines GluN1, GluN2A, GluN2B and GluN2C), CaMKII (Ca2+/calmodulin-dependent protein kinase II - serine in GluN2B), Casein Kinase 2 (serine in GluN2B), and Src kinases (at tyrosines in GluN2A, 2B and 2C) (6, 7). A previous study has reported that reduced expression of GluN2A is associated with age-related working memory impairment, indicating the critical role of GluN2A in working memory (8). On the other hand, the GluN3A subunit is a unique inhibitory subunit among the subunits of NMDA receptors (9). GluN3A decreases Mg^2+^ blockade and Ca^2+^ permeability and augments neuronal excitability at resting potentials (10). GluN3A subunit is expressed in the CNS including the hippocampus and the cortex, and involved in synaptic plasticity (11, 12). GluN3A potently affects dendritic spine density and has a critical role in regulating glutamatergic synaptic plasticity (13).  

Opioids including morphine, the µ-opioid receptor agonist, are widely used for the treatment of moderate to severe pain (14). Behavioral and neuro-adaptive effects of morphine are related to stimulation of NMDA receptors (15). Previous research has suggested that NMDA receptors are involved in morphine tolerance and dependence (16). It has been also reported that morphine increases the expression level of GluN2B in the limbic system (17). Furthermore, administration of a single dose of morphine decreases the expression levels of GluN1, GluN2A, and GluN2B subunits in the hippocampus, 4 hr after administration (18). 

NeuroAid is a traditional Chinese medicine with anti-apoptotic and anti-inflammatory effects (19). A previous study has declared that NeuroAid treatment improves animal survival as well as functional neurological recovery and attenuates neurodegeneration (20). NeuroAid also prevents neuronal necrosis and apoptosis (21). NeuroAid has a significant protective effect against glutamate-induced cell death (20). It seems that NeuroAid by activating BDNF (brain-derived neurotrophic factor) modulates neuronal survival and exerts a neuroprotective effect against glutamate-induced damages (22). The inhibitory effect of NeuroAid on glutamate-induced excitotoxicity has been also revealed in other studies (20, 21). 

According to the points mentioned, the goal of this study is to investigate the effects of morphine and NeuroAid on the expression levels of GluN2A and GluN3A in the hippocampus and striatum of male Wistar rats.

## Materials and Methods

***Animals***

Twenty male Wistar rats (220–240 g) were obtained from the Pasteur Institute. The rats were randomly housed in groups of five in each Plexiglas cage and maintained under controlled environmental conditions including stable light/dark cycle (7:00-19:00 lights on) and temperature (22±2 °C). All rats had *ad libitum* access to water and food. Our experimental protocol was done in accord with the Institute for Cognitive Science Studies (ICSS) guidelines and the National Institutes of Health Guide for the Care and Use of Laboratory Animals (NIH publications No. 80–23).

***Drugs***

Morphine sulfate (Temad company, Tehran, Iran) and NeuroAid (MLC601, Moleac company,  Singapore) were used in this research. Both drugs were dissolved in normal saline and injected intraperitoneally (IP). NeuroAid consisted of nine herbal ingredients (extracts of Radix astragali, Radix salvia miltiorrhizae, Radix paeoniae rubra, Rhizoma chuanxiong, Radix angelicae sinensis, Carthamus tinctorius, Prunus persica, Radix polygalae, and Rhizoma acori tatarinowii) and five non-herbal components (Hirudo, Eupolyphaga seu steleophaga, Calculus bovis artifactus, Buthus martensii and Cornu saigae tataricae).  

***Experimental design***

**Figure 1:**
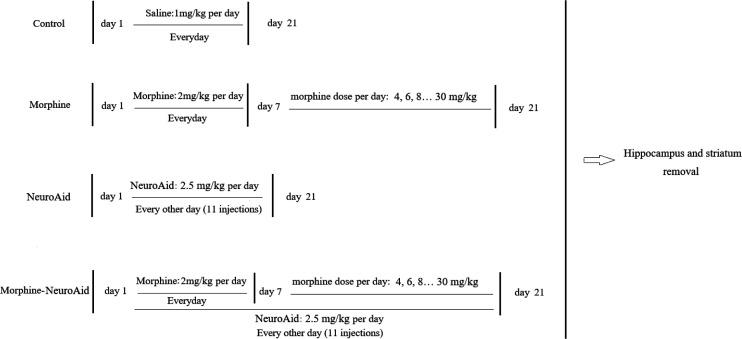
The time-line of the study that shows the schedule of the injections. Twenty rats were randomly divided into four groups: control, morphine, NeuroAid, and morphine-NeuroAid (each group, five rats)

Twenty rats were randomly divided into four groups: control, morphine, NeuroAid, and morphine-NeuroAid (five rats/group). Rats in the control group received saline without any kind of drug intervention for 21 days. Rats in the morphine group received a fixed dose of morphine (2 mg/kg) for 7 days, and then, during 14 days, the rats received morphine at increasing doses (2 mg/kg per day) until the last day (4, 6, 8, 10, …, 30 mg/kg until day 21). Rats in the NeuroAid group received NeuroAid (2.5 mg/kg) every other day until day 21. Rats in the morphine-NeuroAid group received morphine plus NeuroAid as did the rats in the morphine and NeuroAid groups, respectively, for 21 days. After 21 days, the brains of all rats were separated and prepared for molecular analysis (Figure 1).

***Tissue sample collection***

Rats were euthanized by decapitation. Following the decapitation, the hippocampus and striatum were removed from the skull, washed with saline, and dissected on a plate full of ice. The hippocampus was separated from the other parts of the brain by cutting the brain’s midline which leads to the separation of the cerebral hemispheres. Then, with the assistance of a spatula, we cut the fimbria-fornix bundle resulting in easier hippocampus dissection.

***RNA isolation and RT-PCR***

The samples were immediately lysed and the RNA extraction procedure was done by a high pure tissue RNA extraction kit (Qia gene) in accordance with its relevant method. Next, the quality of the extracted RNA was checked by NanoDrop spectrophotometers. c-DNA was synthesized by reverse transcription kit (Qia gene). Following this step, real-time polymerase chain reaction was performed as a rapid method for the amplification of c-DNA.

***Oligonucleotide set design***

 In this study, GluN2A and GluN3A were selected as targets and beta-actin was selected as the internal reference gene. The sequences of these genes were obtained from the NCBI database and primer sets were designed using the GeneRunner software package and analyzed in Basic Local Alignment Search Tool to avoid secondary structure and homology with other genome regions (Table 1).

***Real-time polymerase chain reaction***

cDNAs were diluted 1 to 5 with distilled water. Therefore, the final cDNA concentration for the PCR reaction was 15 ng/ μl. Then, all the primers were diluted to a concentration of 0.2 pmol/μl. At the end, Master Mix (RealQ Plus 2x Master Mix Green, AMPLIQON, Denmark), cDNA, and primers in the 96 wells of Applied Biosystems (USA StepOnePlus Real-Time PCR) were added, and at this stage, done according to the Master Mix protocol (Table 1). Plate design was carried out using two repetitions of each sample to increase accuracy. Primary denaturation was carried out at 95 °C for 15 min and 40 cycles, including denaturation at 95 °C for 15 sec and the annealing phase at 64 °C for 60 sec. Relative gene expression levels were calculated by the Pfaffl method. For drawing standard curves proliferation, at first, the cDNAs of the control group mixed and dilutions of 0.6, 3, 15, and 75 ng/μl were prepared. Then, real-time PCR reaction was repeated twice, for each dilution with each of the primers individually. In the end, the standard curve for each primer, based on the obtained values of Ct in contrast to the used dilutions, was drawn. Using the obtained curve line gradient and this relation “E = 10 (-1/slope) – 1” (E: reaction efficiency, slope: curve line gradient), reaction efficiency for each primer was measured.

***Statistical analysis***

Statistical analyses were performed using SPSS software (V. 22.0). One-way ANOVA and *post hoc* Tukey tests were used to assess the significant difference between groups. *P*<0.05 was considered statistically significant. 

## Results

***Effect of morphine and NeuroAid on the expression level of GluN2A and GluN3A in the hippocampus***

**Figure 2:**
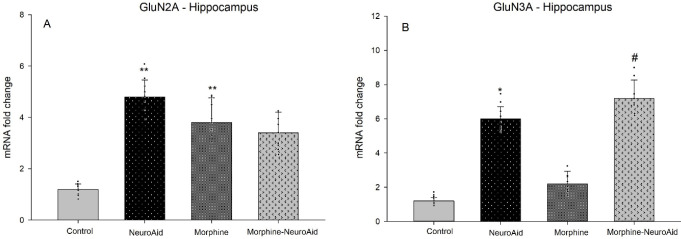
The expression levels of GluN2A and GluN3A in the hippocampus following administration of morphine and NeuroAid [***P<0.001, **P<0.01, *P<0.05 compared with the control group, ###*P*<0.001, ##*P*<0.01, #*P*<0.05 compared with the morphine group]

The results showed that morphine (95% CIs 3.50 to 4.68, *P*=0.004) and NeuroAid (95% CIs 4.31 to 5.46, *P*=0.002) up-regulated the expression level of GluN2A in the hippocampus (Figure 2, Panel A). Also, only NeuroAid (95% CIs 5.54 to 6.91, *P*=0.028) up-regulated the expression level of GluN3A in the hippocampus (Figure 2, Panel B). Moreover, NeuroAid (95% CIs 6.44 to 8.20, *P*=0.021) increased the expression level of GluN3A in the hippocampus of morphine-addicted rats (Figure 2, Panel B).

***The effect of morphine and NeuroAid on the expression level of GluN2A and GluN3A in the striatum***

**Figure 3:**
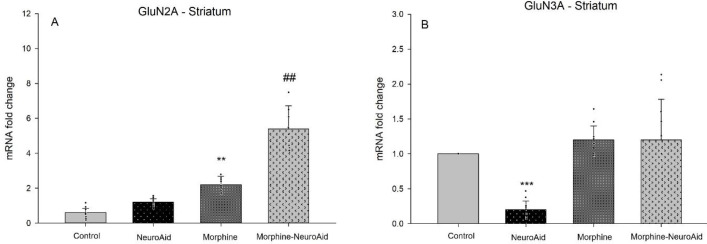
The expression level of GluN2A and GluN3A in the striatum following administration of morphine and NeuroAid [***P<0.001, **P<0.01, *P<0.05 compared with the control group, ###*P*<0.001, ##*P*<0.01, #*P*<0.05 compared with the morphine group]

The results showed that morphine (95% CIs 1.96 to 2.76, *P*=0.008) up-regulated the expression level of GluN2A in the striatum (Figure 3, Panel A). Also, NeuroAid (95% CIs 4.64 to 6.68, *P*=0.002) increased the expression level of GluN2A in the striatum of morphine-addicted rats (Figure 3, Panel A). Moreover, NeuroAid (95% CIs 0.16 to 0.39, *P*=0.000) down-regulated the expression level of GluN3A in the striatum (Figure 3, Panel B). 

**Table 1:** Characteristics of the primers used in the Real-time PCR assay. GluN2A and GluN3A were selected as targets and beta-actin was selected as internal reference gene

	*Sequences*	*Gene*
**F: 22** **R: 20**	F: GCCATGGATGACGATATCGCTG R: CCCATACCCACCATCACACC	*Beta-actin*
**F: 22** **R: 24**	F: TGCATCTATGATCATGGCTGAC R: ATAAAGCTGATGAAGTCTCGGTAG	*GluN2A*
**F: 19** **R: 20**	F: AACCTCAAGCGCATCGGAC R: GTGCCGGACTCTGGATCATC	*GluN3A*

## Discussion

***The effect of morphine on the expression level of GluN2A and GluN3A in the hippocampus and striatum***

As the results showed, morphine up-regulated the expression of GluN2A in both brain regions, with no effect on the expression of GluN3A. Morphine is derived from *Papaver somniferum* and used for the treatment of pain (23). Different studies have mentioned the interaction of morphine and NMDA receptors (24, 25). It has been reported that GluN2A subunits have a critical role in learning and memory. Previous research has demonstrated that mice lacking hippocampal GluN2A subunits show spatial working memory impairment (26). Another study has reported an increase in GluN2A subunits at dendritic spines after the induction of LTP in the hippocampus (27). Blocking GluN2A subunits in the basolateral amygdala impairs reconsolidation of conditioned fear memory (28). On the other hand, NMDA receptor antagonists prevent morphine-induced CPP (Conditioned Place Preference) in mice (29), while NMDA receptor agonists facilitate morphine-induced CPP (30). Polyamines, naturally occurring polycations that activate GluN2B subunits, bind at a dimer interface between GluN1 and GluN2B subunits. It has been revealed that arcaine (antagonist of the NMDA receptor polyamine-binding site) interacts with morphine-induced behavioral alterations (31, 32). It’s important to note that, GluN2A subunits are involved in morphine dependence (33). Previous research has declared that the signs of morphine withdrawal are significantly decreased in GluN2A knockout mice. These results suggested that GluN2A-induced adaptive alterations have a key role in the development of morphine dependence (34). Additionally, up-regulated GluN2A expression is observed in the nucleus accumbens of mice after chronic morphine dependence (35). It has been also suggested that opioidergic neurons are connected to the glutamatergic neurons and activate postsynaptic GluN2A subunits via unknown inhibitory neurons because the morphine analgesic effect is potent in GluN2A^-/-^ mice (35). Moreover, the expression level of GluN2A is significantly increased after chronic treatment of morphine in PAG (periaqueductal gray), VTA (ventral tegmental area), and NAc (nucleus accumbens shell), indicating the role of GluN2A subunits in the development of morphine analgesic tolerance (35). Previous research has also revealed that activation of D1 dopaminergic receptors regulates morphine-induced LTP by direct interaction with GluN2A subunits at a wide-range of synapses from the prefrontal cortex to the NAc and plays a significant role in addiction-related synaptic plasticity changes (36). In line with these findings, our data showed that morphine up-regulated the expression level of GluN2A in the hippocampus and striatum. These results may reveal the important role of hippocampal and striatal GluN2A subunits in morphine analgesic tolerance, morphine physical dependence, and morphine reward-related memory. Behavioral experiments are needed to better understand the interaction effect of GluN2A and morphine in the striatum and the hippocampus.

***The effect of NeuroAid on the expression level of GluN2A and GluN3A in the hippocampus and striatum***

As the results showed, NeuroAid up-regulated the expression of both subunits in the hippocampus, while down-regulating the expression of GluN3A in the striatum. NeuroAid (MLC601) is a Chinese drug with nine herbal ingredients and five non-herbal components (37). NeuroAid has neuroprotective and anti-apoptotic effects (20). Furthermore, NeuroAid modulates neuronal survival and exerts a neuroprotective effect against glutamate-induced damages (22). In addition, NeuroAid improves functional neurological recovery and attenuates neurodegeneration (20). Previous research has shown that post-treatment administration of NeuroAid for one week significantly attenuates neuronal death in the CA1 hippocampal region (21). NeuroAid also induces a potent protective effect against glutamate-induced cell death (20). It should be noted that there are not many published papers about the effect of NeuroAid on the expression of GluNs subunits. Also, the mechanism of NeuroAid is largely unknown; however, the neuroprotective effect of NeuroAid has been reported, especially in stroke and traumatic brain injuries. Previous research has reported that NeuroAid improves cognitive functions including episodic-like memory after traumatic brain injury (38). NeuroAid also promotes extinction in passive avoidance tasks and improves learning in the Morris Water Maze apparatus, and also, enhances the performance of mice in the novel object recognition test. NeuroAid increases the number of hippocampal mature neurons (39). On the other hand, the positive role of GluN2A in synaptic plasticity and memory has been revealed. After the induction of synaptic plasticity, GluN2A subunits are increased in the synaptic fraction (40). After effective LTP induction via theta-burst stimulation (TBS), GluN2A subunits are increased in the hippocampus (41). Furthermore, mice lacking hippocampal GluN2A subunits show disrupted spatial working memory (26). As mentioned, GluN3A affects dendritic spine density and regulates glutamatergic synaptic plasticity (13). Astrocytes play a role in the regulation of synaptic transmission and LTP induction via releasing glutamate. Furthermore, astrocytes affect intracellular Ca^2+^ homeostasis via modulating GluN3A expression (42, 43). GluN3A is a negative subunit, that constrains NMDA-induced calcium influx (44). Also, NMDA activation in GluN3A knockout mice is increased (1). According to these reports, we can suggest that NeuroAid-induced hippocampal GluN2A up-regulation which induces LTP and synaptic plasticity, may be related to its improvement effect on learning and memory. We can also suggest that NeuroAid-induced striatal GluN3A down-regulation may be related to its neuroprotective and anti-apoptotic effects. This mechanism may be associated with the protective effect of NeuroAid on glutamate-induced cell death and excitotoxicity. It can be also suggested that the therapeutic effect of NeuroAid on Parkinson’s disease, in addition to its anti-inflammatory effect, may be related to its anti-apoptotic effect in the striatum. 

***The effect of NeuroAid on the expression level of GluN2A and GluN3A in the hippocampus and striatum of morphine-addicted rats***

As the results showed, NeuroAid up-regulated the expression level of GluN3A in the hippocampus and GluN2A in the striatum of morphine-addicted rats. It should be noted that this is the first research about the effect of interaction between NeuroAid and morphine on the expression level of NMDA GluNs subunits. As we mentioned, GluN3A subunits are negative subunits of NMDA receptors that prevent NMDA-induced calcium influx. Also, we mentioned that GluN3A knocking out leads to enhanced NMDA current. It has been revealed that morphine induces apoptosis (45). Furthermore, morphine administration leads to apoptotic effects in the heart tissue of mice (46). Stress-induced reinstatement of morphine-induced CPP is related to increase in glutamate in the medial prefrontal cortex (47). Also, chronic opioid exposure leads to glutamatergic neurotransmission adaptation which has a significant role in the modulation of addiction (48). Previous research has revealed that Agmatine by regulating the release of glutamate and expression of GluNs, decreases the extracellular level of glutamate, which in turn leads to attenuated morphine-induced glutamatergic adaptation in the hippocampus (48). We suggest that up-regulation of GluN3A subunits in the hippocampus may be a neuroprotective mechanism of NeuroAid to cope with morphine-induced cell death and morphine-induced addiction. These results suggest that NeuroAid may interact with the effect of morphine on glutamatergic neurotransmission. On the other hand, NeuroAid increased the expression of GluN2A in the striatum of morphine-addicted rats. There is no published data on this topic and to better understand the interaction effect between morphine and NeuroAid, further studies are needed. We can suggest that NeuroAid by altering the expression of GluN2A in morphine-addicted rats affects the development of morphine analgesic tolerance. 

## Conclusion

The results of this study showed that morphine increased the expression level of GluN2A in the hippocampus and striatum. NeuroAid increased the expression level of GluN2A and GluN3A in the hippocampus, while decreasing the expression of GluN3A in the striatum. NeuroAid also increased the expression of GluN3A in the hippocampus and GluN2A in the striatum of morphine-addicted rats. It should be noted that there is no published paper in this field and to better understand the interaction effect of NeuroAid and morphine on the expression of GluN2A and GluN3A in the hippocampus and striatum, further studies are needed.

## References

[1] Tong G, Takahashi H, Tu S, Shin Y, Talantova M, Zago W (2008). Modulation of NMDA receptor properties and synaptic transmission by the NR3A subunit in mouse hippocampal and cerebrocortical neurons. J Neurophysiol.

[2] Traynelis SF, Wollmuth LP, McBain CJ, Menniti FS, Vance KM, Ogden KK (2010). Glutamate receptor ion channels: structure, regulation, and function. Pharmacol Rev.

[3] Grand T, Abi Gerges S, David M, Diana MA, Paoletti P (2018). Unmasking GluN1/GluN3A excitatory glycine NMDA receptors. Nat Commun.

[4] Sanz-Clemente A, Nicoll RA, Roche KW (2013). Diversity in NMDA receptor composition: many regulators, many consequences. Neuroscientist.

[5] Zhou C, Sun H, Klein PM, Jensen FE (2015). Neonatal seizures alter NMDA glutamate receptor GluN2A and 3A subunit expression and function in hippocampal CA1 neurons. Front Cell Neurosci.

[6] Sanz-Clemente A, Gray JA, Ogilvie KA, Nicoll RA, Roche KW (2013). Activated CaMKII couples GluN2B and casein kinase 2 to control synaptic NMDA receptors. Cell Rep.

[7] Zhou Y, Takahashi E, Li W, Halt A, Wiltgen B, Ehninger D (2007). Interactions between the NR2B receptor and CaMKII modulate synaptic plasticity and spatial learning. J Neurosci.

[8] McQuail JA, Beas BS, Kelly KB, Simpson KL, Frazier CJ, Setlow B (2016). NR2A-containing NMDARs in the prefrontal cortex are required for working memory and associated with age-related cognitive decline. J Neurosci.

[9] Lee JH, Wei L, Deveau TC, Gu X, Yu SP (2016). Expression of the NMDA receptor subunit GluN3A (NR3A) in the olfactory system and its regulatory role on olfaction in the adult mouse. Brain Struct Funct.

[10] Henson MA, Roberts AC, Perez-Otano I, Philpot BD (2010). Influence of the NR3A subunit on NMDA receptor functions. Prog Neurobiol.

[11] Larsen RS, Corlew RJ, Henson MA, Roberts AC, Mishina M, Watanabe M (2011). NR3A-containing NMDARs promote neurotransmitter release and spike timing-dependent plasticity. Nat Neurosci.

[12] Pachernegg S, Strutz-Seebohm N, Hollmann M (2012). GluN3 subunit-containing NMDA receptors: not just one-trick ponies. Trends Neurosci.

[13] Yuan T, Bellone C (2013). Glutamatergic receptors at developing synapses: the role of GluN3A-containing NMDA receptors and GluA2-lacking AMPA receptors. Eur J Pharmacol.

[14] Ko SW, Wu LJ, Shum F, Quan J, Zhuo M (2008). Cingulate NMDA NR2B receptors contribute to morphine-induced analgesic tolerance. Mol Brain.

[15] Jeziorski M, White FJ, Wolf ME (1994). MK-801 prevents the development of behavioral sensitization during repeated morphine administration. Synapse.

[16] Ma YY, Chu NN, Guo CY, Han JS, Cui CL (2007). NR2B-containing NMDA receptor is required for morphine-but not stress-induced reinstatement. Exp Neurol.

[17] Bajo M, Crawford EF, Roberto M, Madamba SG, Siggins GR (2006). Chronic morphine treatment alters expression of N-methyl-D-aspartate receptor subunits in the extended amygdala. J Neurosci Res.

[18] Le Greves P, Huang W, Zhou Q, Thornwall M, Nyberg F (1998). Acute effects of morphine on the expression of mRNAs for NMDA receptor subunits in the rat hippocampus, hypothalamus and spinal cord. Eur J Pharmacol.

[19] Chan HY, Stanton LW (2016). A pharmacogenomic profile of human neural progenitors undergoing differentiation in the presence of the traditional Chinese medicine NeuroAiD. Pharmacogenomics J.

[20] Heurteaux C, Gandin C, Borsotto M, Widmann C, Brau F, Lhuillier M (2010). Neuroprotective and neuroproliferative activities of NeuroAid (MLC601, MLC901), a Chinese medicine, in vitro and in vivo. Neuropharmacology.

[21] Quintard H, Borsotto M, Veyssiere J, Gandin C, Labbal F, Widmann C (2011). MLC901, a traditional Chinese medicine protects the brain against global ischemia. Neuropharmacology.

[22] Mattson MP (2008). Glutamate and neurotrophic factors in neuronal plasticity and disease. Ann N Y Acad Sci.

[23] Laux-Biehlmann A, Mouheiche J, Veriepe J, Goumon Y (2013). Endogenous morphine and its metabolites in mammals: history, synthesis, localization and perspectives. Neuroscience.

[24] Tomazi L, Mello CF, Schoffer AP, Girardi BA, Fruhauf PK, Rubin MA (2017). A Nonrewarding NMDA Receptor Antagonist Impairs the Acquisition, Consolidation, and Expression of Morphine Conditioned Place Preference in Mice. Mol Neurobiol.

[25] Lilius TO, Viisanen H, Jokinen V, Niemi M, Kalso EA, Rauhala PV (2018). Interactions of (2S,6S;2R,6R)-hydroxynorketamine, a secondary metabolite of (R,S)-ketamine, with morphine. Basic Clin Pharmacol Toxicol.

[26] Bannerman DM, Niewoehner B, Lyon L, Romberg C, Schmitt WB, Taylor A (2008). NMDA receptor subunit NR2A is required for rapidly acquired spatial working memory but not incremental spatial reference memory. J Neurosci.

[27] Barria A, Malinow R (2002). Subunit-specific NMDA receptor trafficking to synapses. Neuron.

[28] Milton AL, Merlo E, Ratano P, Gregory BL, Dumbreck JK, Everitt BJ (2013). Double dissociation of the requirement for GluN2B- and GluN2A-containing NMDA receptors in the destabilization and restabilization of a reconsolidating memory. J Neurosci.

[29] Lue WM, Huang EY, Yang SN, Wong CS, Tao PL (2007). Post-treatment of dextromethorphan reverses morphine effect on conditioned place preference in rats. Synapse.

[30] Zarrindast MR, Lashgari R, Rezayof A, Motamedi F, Nazari-Serenjeh F (2007). NMDA receptors of dorsal hippocampus are involved in the acquisition, but not in the expression of morphine-induced place preference. Eur J Pharmacol.

[31] Mony L, Zhu S, Carvalho S, Paoletti P (2011). Molecular basis of positive allosteric modulation of GluN2B NMDA receptors by polyamines. EMBO J.

[32] Mariani RK, Mello CF, Rosa MM, Ceretta AP, Camera K, Rubin MA (2011). Effect of naloxone and morphine on arcaine-induced state-dependent memory in rats. Psychopharmacology (Berl).

[33] Paoletti P (2011). Molecular basis of NMDA receptor functional diversity. Eur J Neurosci.

[34] Miyamoto H, Rahman MM, Chang C (2004). Molecular basis for the antiandrogen withdrawal syndrome. J Cell Biochem.

[35] Inoue M, Mishina M, Ueda H (2003). Locus-specific rescue of GluRepsilon1 NMDA receptors in mutant mice identifies the brain regions important for morphine tolerance and dependence. J Neurosci.

[36] Zheng Q, Liu Z, Wei C, Han J, Liu Y, Zhang X (2014). Activation of the D1 receptors inhibits the long-term potentiation in vivo induced by acute morphine administration through a D1-GluN2A interaction in the nucleus accumbens. Neuroreport.

[37] Heurteaux C, Widmann C, Moha ou Maati H, Quintard H, Gandin C, Borsotto M (2013). NeuroAiD: properties for neuroprotection and neurorepair. Cerebrovasc Dis.

[38] Quintard H, Lorivel T, Gandin C, Lazdunski M, Heurteaux C (2014). MLC901, a Traditional Chinese Medicine induces neuroprotective and neuroregenerative benefits after traumatic brain injury in rats. Neuroscience.

[39] Lorivel T, Gandin C, Veyssiere J, Lazdunski M, Heurteaux C (2015). Positive effects of the traditional Chinese medicine MLC901 in cognitive tasks. J Neurosci Res.

[40] Grosshans DR, Clayton DA, Coultrap SJ, Browning MD (2002). LTP leads to rapid surface expression of NMDA but not AMPA receptors in adult rat CA1. Nat Neurosci.

[41] Baez MV, Oberholzer MV, Cercato MC, Snitcofsky M, Aguirre AI, Jerusalinsky DA (2013). NMDA receptor subunits in the adult rat hippocampus undergo similar changes after 5 minutes in an open field and after LTP induction. PLoS One.

[42] Lee MC, Ting KK, Adams S, Brew BJ, Chung R, Guillemin GJ (2010). Characterisation of the expression of NMDA receptors in human astrocytes. PLoS One.

[43] Kehoe LA, Bernardinelli Y, Muller D (2013). GluN3A: An NMDA receptor subunit with exquisite properties and functions. Neural Plast.

[44] Rozeboom AM, Queenan BN, Partridge JG, Farnham C, Wu JY, Vicini S (2015). Evidence for glycinergic GluN1/GluN3 NMDA receptors in hippocampal metaplasticity. Neurobiol Learn Mem.

[45] Lou W, Zhang X, Hu XY, Hu AR (2016). MicroRNA-219-5p inhibits morphine-induced apoptosis by targeting key cell cycle regulator WEE1. Med Sci Monit.

[46] Jalili C, Sohrabi M, Jalili F, Salahshoor MR (2018). Assessment of thymoquinone effects on apoptotic and oxidative damage induced by morphine in mice heart. Cell Mol Biol.

[47] Liu P, Che X, Yu L, Yang X, An N, Song W (2017). Uridine attenuates morphine-induced conditioned place preference and regulates glutamate/GABA levels in mPFC of mice. Pharmacol Biochem Behav.

[48] Wang XF, Zhao TY, Su RB, Wu N, Li J (2016). Agmatine prevents adaptation of the hippocampal glutamate system in chronic morphine-treated rats. Neurosci Bull.

